# JobCenter: an open source, cross-platform, and distributed job queue management system optimized for scalability and versatility

**DOI:** 10.1186/1751-0473-7-8

**Published:** 2012-07-30

**Authors:** Daniel Jaschob, Michael Riffle

**Affiliations:** 1 Department of Biochemistry, University of Washington, 1705 NE Pacific St, Box 357350, Seattle, WA, 98195, USA; 2 Department of Genome Sciences, University of Washington, Seattle, WA, 98195, USA

**Keywords:** Computing, Bioinformatics, Computational biology, Job management, Distributed, Open-source, Cross-platform, Grid computing, Job scheduler, Batch processing

## Abstract

**Background:**

Laboratories engaged in computational biology or bioinformatics frequently need to run lengthy, multistep, and user-driven computational jobs. Each job can tie up a computer for a few minutes to several days, and many laboratories lack the expertise or resources to build and maintain a dedicated computer cluster.

**Results:**

JobCenter is a client–server application and framework for job management and distributed job execution. The client and server components are both written in Java and are cross-platform and relatively easy to install. All communication with the server is client-driven, which allows worker nodes to run anywhere (even behind external firewalls or “in the cloud”) and provides inherent load balancing. Adding a worker node to the worker pool is as simple as dropping the JobCenter client files onto any computer and performing basic configuration, which provides tremendous ease-of-use, flexibility, and limitless horizontal scalability. Each worker installation may be independently configured, including the types of jobs it is able to run. Executed jobs may be written in any language and may include multistep workflows.

**Conclusions:**

JobCenter is a versatile and scalable distributed job management system that allows laboratories to very efficiently distribute all computational work among available resources. JobCenter is freely available at http://code.google.com/p/jobcenter/.

## Background

Biomedical research laboratories often have need to run lengthy, computationally intensive bioinformatics and computational biology applications. These applications may include items such as finding sequence homologs using BLAST [1]; running proteomics search algorithms, such as Mascot [2], SEQUEST [3], or PeptideProphet [4]; statistically analyzing and producing visual outputs for large, complex datasets; or automatically executing multistep workflows that take raw data all the way to post-processed visualization via a database-driven web site. Often these applications are installed in a non-standard *ad hoc* manner on laboratory computers, and users log into these machines to manually execute the software as needed. Not only does this require continual training of new lab members (as to the location, interface, and quirks of particular software), it is an inefficient use of laboratory resources. Relatively few of the available computers will carry the majority of the computational burden, and in the case of web-driven applications, the execution will potentially tie up the web server itself--limiting performance, resource availability, and possibly crashing the server itself. Ideally, execution of these applications would take place on dedicated specially-configured hardware, such as computer clusters; however many laboratories may lack the resources to dedicate hardware exclusively to the execution of these applications, let alone the expertise and resources required to design, install, and maintain a computer cluster.

Here we present JobCenter, a conceptually simple software package for managing the execution of computational jobs in a distributed computing environment. It uses an implementation of a client server model for job management, where the clients request work from the server and the server then decides which job to send based on queued jobs, their priorities, and the client’s reported capabilities. Because the client is written in Java and is cross-platform, JobCenter may effectively utilize all computational resources available in the heterogeneous “organically grown” computing environments typically found in laboratories. To turn a computer into a worker, simply install the client and perform basic configuration. Because the client initiates communication, it may exist on any machine capable of communicating with the server--whether it’s on the same local network, behind an external firewall, or a virtual server “in the cloud”. This provides JobCenter with significant horizontal scalability. Both the client and server are relatively easy to install, and a simple graphical user interface for viewing and managing jobs is included. JobCenter is open-source and freely available at http://code.google.com/p/jobcenter/.

## Implementation

### Overall architecture

**Figure 1:**
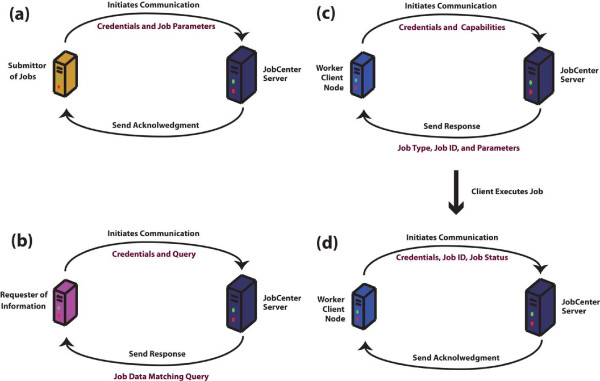
**A depiction of the client-pull architecture of JobCenter and four possible types of client-initiated communication with the JobCenter server.** (**a**) Software responsible for submitting jobs to the JobCenter server initiate a connection with the server, communicating authentication credentials and data describing the job. (**b**) Software interested in the data in the job queue database, such as a GUI for managing the database, communicate with the server sending requests for data. (**c**) Worker nodes (computers with the JobCenter client installed) periodically initiate a connection with the server requesting work. (**d**) Upon completion of the work, the client initiates a connection with the server to communicate the status of the job and any messages that resulted from execution.

JobCenter implements a distributed client–server architecture using client pull communication, where communication is never initiated by the server (Figure 1). All communication with the server takes place over standard HTTP using REST web services and XML.

### Client

**Figure 2:**
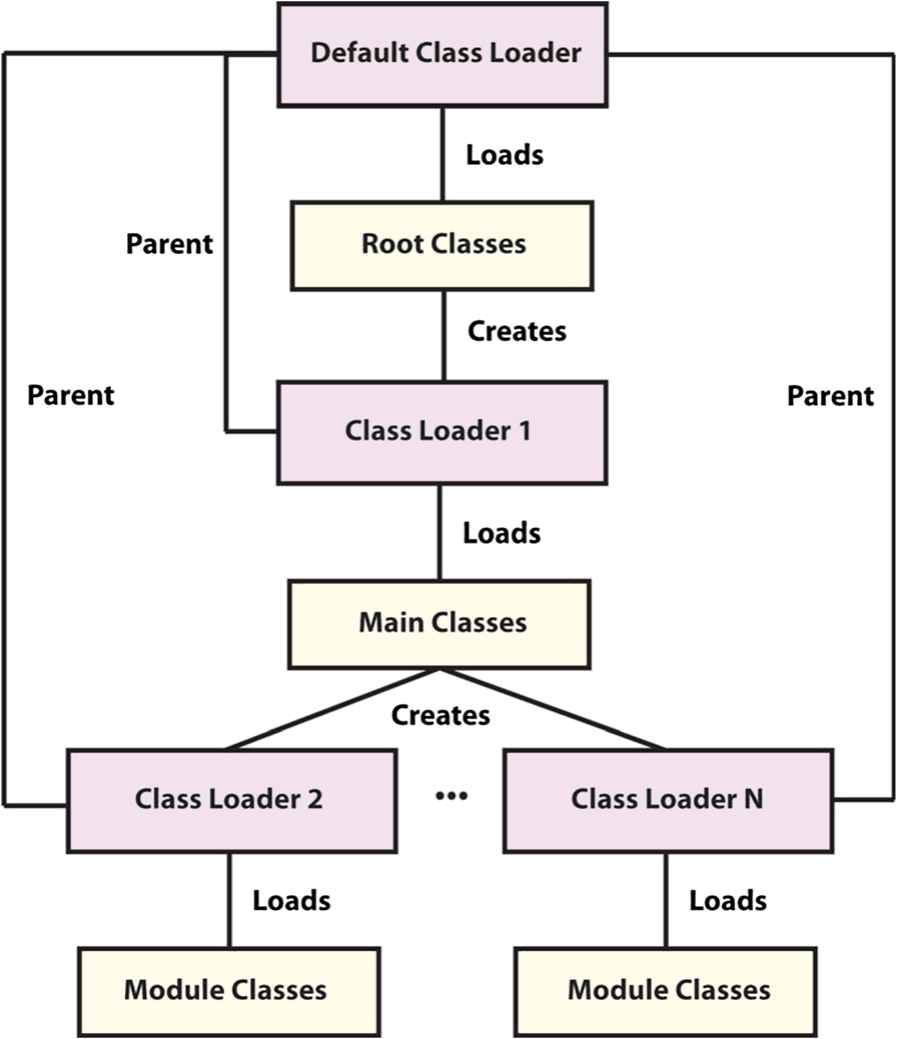
**JobCenter leverages Java class loaders to separately load modules with their own namespaces to avoid library, package, class, or interface collisions with the main JobCenter classes or other modules.** When executed, JobCenter loads a minimal set of root classes and a single external library that are primarily responsible for loading the main classes using a new class loader. These main classes contain the majority of code and libraries necessary for running JobCenter and are responsible for loading modules using a new class loader for each module. Typically, classes loaded in this manner would share namespace with their parent class loader (main), but the parent class loader for modules is explicitly set to be the root class loader. This separates module namespaces from each other, as well as from the large set of main classes and libraries that run JobCenter.

The client is written in the Java programming language and may be run on any platform where version 1.6 or later of the Sun Java Runtime Environment (JRE) is available. The client is organized as a core set of Java classes and one or more user-created modules. A module is a set of Java classes adhering to the module interface defined by JobCenter, and is responsible for the execution of a particular type of job (e.g., running BLAST). Modules are essentially Java wrappers for the execution of programs (which may or may not be written in Java) that adhere to this interface. Each module is loaded using separate Java class loaders in order to avoid namespace collisions of classes or Java libraries used by individual modules or by JobCenter itself (Figure 2). Each module is deployed as a separate directory in the modules directory of a JobCenter installation and are detected at start up and subsequently communicated to the server as the types of jobs the client is capable of executing.

**Figure 3:**
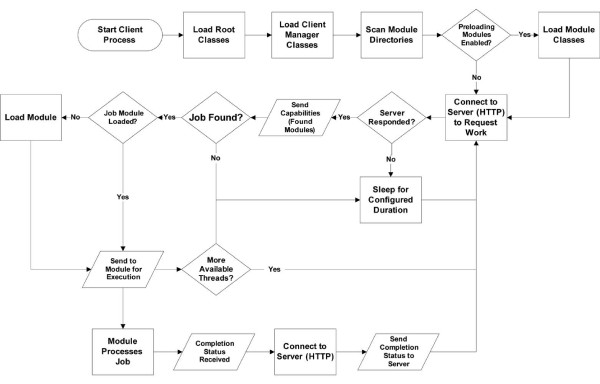
A flow chart depicting the start up and ongoing behavior and decision-making of the JobCenter client with regards to sleeping, requesting work, and processing work.

The client follows a cycle of sleeping (configurable duration), requesting work, executing work, reporting status to server, and requesting more work (Figure 3). The client initiates contact with the server via web services to request work, communicating its unique identifier string (used for authentication, see “Security” below) and the types jobs it is configured to run (modules present at startup). Which jobs are returned for execution is solely at the discretion of the server. Each job runs in a separate thread, and the number of job threads the client may run simultaneously is configurable in the client’s properties file. Upon completion of the job, the client reinitiates contact with the server reporting the status (success or error), messages, and other data relevant to job completion to the server.

Clients also establish periodic connections via web services to the server solely to report that they are still up and running and that they will report in again in a set number of seconds (configurable at the client level). If a client fails to report to the server after this time has elapsed, the server may respond by triggering an alarm that sends an email to the administrator (configurable at the server level).

### Server

The server is written in Java and implemented as a Java web application meant to be run using a Java servlet container, such as Apache Tomcat. Accordingly, the server is cross platform and relatively simple to install--a matter of deploying a Web application ARchive (WAR) file to the appropriate directory and performing configuration. The server exposes methods for performing all necessary operations using REST web services and XML. These methods include submitting new jobs; requesting jobs for work; returning lists of pending, running, and completed jobs; updating jobs statuses; and so forth. The full list of interfaces, options, and formats is available in the documentation on the source code repository.

On the backend, the server interfaces with a relational database management system (RDBMS) to interact with job queue data. As currently configured, JobCenter uses the MySQL RDBMS, and all SQL necessary to create the jobs database is available at the source code repository.

### Graphical user interface

JobCenter includes a simple implementation of a graphical user interface (GUI), the functionality of which is described in more detail in “RESULTS AND DISCUSSION.” The GUI is written in Java and implemented as a Java web application meant to be run using a Java servlet container, such as Apache Tomcat. The GUI is entirely independent from the server component of JobCenter, and interacts with the server purely through web services. A custom-developed GUI could conceivably be developed with moderate effort that utilized the same web services and improved the interface for specific needs or to display specific information.

### Security

Authentication is based on unique identifier strings configured in the properties file of each client and the IP addresses from which those unique string identifiers may connect. This prevents unauthorized access to viewing, submitting, or, changing job information in the database. The pairing of unique strings and IP addresses is configured in the database in the “node_access_rule” table.

## Results and discussion

Please note that more detailed instructions regarding installing, configuring, and using JobCenter are available at the JobCenter source code web site at http://code.google.com/p/jobcenter/.

### Using JobCenter

In order for JobCenter to begin executing a new type of job, several steps must be undertaken. If not already done, the JobCenter server must be installed and configured on a computer that may be reached via a network from all client nodes. An entry must be made for the new job type in the JobCenter database, which includes a unique identifier string for that job type, the minimum version required to execute that job (prevents worker nodes with obsolete versions from executing the job), and priority of that job so the server may send out the most important work first. Additionally, code must be added to an existing application that will submit requests to perform the work to the JobCenter server. Once this is done a module must be developed for executing this type of job.

A module is a set of Java classes adhering to the JobCenter module interface that operate as a wrapper around any program that needs to be run. Modules may be responsible for executing any computational operation, such as Perl scripts, R or Stata scripts, Matlab programs, Java programs, compiled binaries, and so on. The JobCenter distribution includes a premade module for executing any program from the command line of Linux or UNIX; however, the execution of more complex programs may require basic Java programming to develop a module to act as a wrapper for the program. Once the module is developed, it must be installed on a client node in order for the job to be executed.

A client node is any computer on which the JobCenter client software has been installed, configured, and is running. Creating a new client node, then, is a matter of installing, configuring, and running the client software on any computer. Installing a module on existing client nodes consists of placing the Java classes for a module in a subdirectory of the modules directory in the client installation and restarting the client. All installed modules are automatically detected on client start up. Once done, the client will immediately be capable of processing jobs of the new type.

### Graphical user interface

A simple graphical user interface (GUI) is available with the JobCenter that provides basic functionality for viewing and managing jobs submitted to a JobCenter server. From this interface users may list all pending, completed, and failed jobs. Jobs may be viewed independently or grouped together by requests. A request in JobCenter is an umbrella for multiple jobs, which constitute independent steps in the single execution of a multistep workflow, or job chain.

From this list users may view specific jobs, where they may see the job parameters and job status (e.g., pending, running, stalled, failed). For pending or failed jobs, users may cancel the job. And for failed jobs, users may requeue the job. Each attempted execution of a specific job in JobCenter is called a run (jobs that result in errors may be requeued, which results in multiple runs for a job). On this page, users may view all attempted runs for a job in chronological order and their exit status and all error messages logged for the run, if any.

Additionally, the GUI has the option of displaying the results of periodic check-ins by active clients, displaying which clients have checked in, when they last checked in, whether or not they are delinquent in checking in, and which jobs the client is currently running.

The provided GUI provides only basic functionality, but it may be expanded, customized, or entirely rewritten in any programming language in order to better provide the specific needs of an organization. The GUI communicates with the JobCenter server exclusively via web services, and the same web services may be used by any programming language.

### Current usage

JobCenter is currently used to process multiple types of jobs by the Yeast Resource Center (YRC) at the University of Washington in Seattle ( http://www.yeastrc.org/). The server component is installed on the YRC production web server, a multi-core Xeon machine running Apache Tomcat on top of Red Hat Enterprise Linux. On the back end, the server connects to MySQL database. The client has been deployed to eight computers running various distributions of Linux, five of which are rack-mounted blades dedicated solely to running as JobCenter clients and are running behind a firewall external to the JobCenter server.

An example of a service supported by JobCenter is the web-driven prediction of signal peptides and transmembrane helices using the Philius [5] prediction algorithm. On the web site, users may submit protein sequences or entire protein FASTA [6] sequence files to be processed. When this is done, the web server submits a job to the JobCenter server using web services. Fulfilling the request requires multiple phases of execution, the first of which must be carried out on the web server. A JobCenter client on the web server, then, requests work and is delivered the job by the server. The job completes, but submits a new job to the server for the next phase of execution. A JobCenter client on one of the dedicated clients requests work and is delivered the next phase of execution, which includes the actual application of the Philius software to the data.

Other examples of JobCenter in use by the YRC include the upload of tandem mass spectrometry (MS/MS) proteomics data to the database and the upload of fluorescence microscopy data to the YRC Public Image Repository [7]. In both cases, requests are initiated by users of a web site, and the web server submits a job to the JobCenter server for the requests. In the case of uploading MS/MS data, the entire upload process is handled by a single job. The client requests work, receives the job, connects to an external server, downloads the data, parses and inserts the data into a relational database, and notifies the user via email when complete. In the case of uploading fluorescence microscopy data, the request is handled by a chain of jobs executed by JobCenter clients on multiple servers, similar to the Philius process.

Additionally, a module has been developed for BLAST that allows for the integration of BLAST searches into the YRC Public Data Repository [8] web interface, allowing users to find protein annotations and experimental data using sequence-based searches against a multi-organism sequence database containing over 45 million distinct sequences. Search requests are submitted to the JobCenter server and distributed to worker nodes that are configured to execute BLAST jobs. Users may wait for their results in real time or receive an email upon completion.

### Future directions

In addition to the development of new, standardized modules, future development of JobCenter will focus largely on: (1) integrating JobCenter with open-source workflow management systems, and (2) simplifying the deployment of modules to worker client computers. Currently, the module must be deployed to every client computer that will run that type of job and, unless the program to be run is written in Java and deployed as part of the module, the program must be installed on each client computer in precisely the same way. If the module is updated, it must be redeployed to every client computer that runs that type of job. Future work will be devoted to shipping the entire execution environment necessary for the execution of a type of job as part of the server’s response to a client’s request for work. This will alleviate the need to explicitly install the modules on all clients, remove the need to install the program to be executed on the clients, and eliminate the possibility that outdated or incorrectly configured instances of the programs are being executed on the clients.

## Conclusions

JobCenter is a cross-platform job queue manager that is designed to leverage computational resources in the heterogeneous *ad hoc* computing environment typically available to biomedical research laboratories. JobCenter allows for the efficient use of all available hardware resources without the potentially onerous requirements for building and maintaining a separate computer cluster. It is relatively simple to install and configure. New worker machines may be added to the pool by installing the client software on virtually any computer in the world, providing flexibility and nearly limitless horizontal scalability. The client pull communication architecture provides JobCenter with innate load balancing, since only workers with unused capacity ask for more work. And, because of the multithreaded nature of the client, each of these workers capacity may be very efficiently utilized.

## Availability and requirements

Project name: JobCenter

Project home page: http://code.google.com/p/jobcenter

Operating system(s): Platform independent (any Java-capable OS)

Programming language: Java

Other requirements: Java 1.6 or higher, Tomcat 6 or higher

License: Apache 2.0

Any restrictions to use by non-academics: None

## Abbreviations

HTTP: Hypertext Transfer Protocol; XML: Extensible Markup Language; JRE: Java Runtime Environment; WAR: Web Application Archive; RDBMS: Relational Database Management System; SQL: Structured Query Language; GUI: Graphical User Interface; YRC: Yeast Resource Center.

## Competing interests

The authors declare that they have no competing interests.

## Authors’ contributions

MR conceived of and directed the project, designed the basic architecture, and prepared the manuscript. DJ designed the software, performed all programming, set up the source code repository, and prepared user documentation. All authors read and approved the final manuscript.

## References

[B1] Altschul SF Gish W Miller W Myers EW Lipman DJ Basic local alignment search tool J Mol Biol 1990 215 3 403 410 2231712 10.1016/S0022-2836(05)80360-2

[B2] Perkins DN Pappin DJ Creasy DM Cottrell JS Probability-based protein identification by searching sequence databases using mass spectrometry data Electrophoresis 1999 20 18 3551 3567 10.1002/(SICI)1522-2683(19991201)20:18<3551::AID-ELPS3551>3.0.CO;2-2 10612281

[B3] Yates JR 3rd Eng JK Mc Cormack AL Mining genomes: correlating tandem mass spectra of modified and unmodified peptides to sequences in nucleotide databases Anal Chem 1995 67 18 3202 3210 10.1021/ac00114a016 8686885

[B4] Deutsch EW Mendoza L Shteynberg D Farrah T Lam H Tasman N Sun Z Nilsson E Pratt B Prazen B A guided tour of the Trans-Proteomic Pipeline Proteomics 2010 10 6 1150 1159 10.1002/pmic.200900375 20101611 PMC3017125

[B5] Reynolds SM Kall L Riffle ME Bilmes JA Noble WS Transmembrane topology and signal peptide prediction using dynamic bayesian networks PLoS Comput Biol 2008 4 11 e1000213 10.1371/journal.pcbi.1000213 18989393 PMC2570248

[B6] Lipman DJ Pearson WR Rapid and sensitive protein similarity searches Science 1985 227 4693 1435 1441 10.1126/science.2983426 2983426

[B7] Riffle M Davis TN The Yeast Resource Center Public Image Repository: A large database of fluorescence microscopy images BMC Bioinformatics 2010 11 263 10.1186/1471-2105-11-263 20482811 PMC2882934

[B8] Riffle M Malmstrom L Davis TN The Yeast Resource Center Public Data Repository Nucleic Acids Res 2005 33 Database issue D378 D382 15608220 10.1093/nar/gki073 PMC540027

